# Failed reintubation during resuscitation after posterior occipito-cervical spinal fusion: A case report

**DOI:** 10.1097/MD.0000000000035427

**Published:** 2023-10-06

**Authors:** Fan Huang, Qing Zhong, Yan Wang, Yan Weng

**Affiliations:** a Department of Anesthesiology, The People’s Hospital of Jianyang, Jianyang, Sichuan, China.

**Keywords:** case report, failed reintubation, Occipito-cervical spinal fusion, atlantoaxial dislocation

## Abstract

**Rationale:**

Posterior occipito-cervical spinal fusion (OCF) is a common surgical method for atlantoaxial dislocation, but postoperative airway complications may occur. Reintubation has been reported in the past, but the failure of reintubation is rare.

**Patient concerns:**

A 54-year-old woman who underwent OCF due to rheumatoid cervical spondylosis and atlantoaxial dislocation. In the postanesthesia care unit, the patient developed upper respiratory tract obstruction after extubation.

**Diagnoses and interventions:**

It is an upper respiratory tract obstruction due to anatomical changes because of surgery. Emergency airway management was activated, but it did not work.

**Outcomes:**

Emergency reintubation failed, and the patient was finally saved after tracheotomy.

**Lessons:**

For patients who underwent OCF due to cervical spondylosis caused by rheumatoid arthritis, great attention should be paid to the perioperative airway management, especially during the recovery period. And more important, adequate reintubation preparatory work should be done before extubation.

## 1. Introduction

Atlantoaxial dislocation (AAD) accounts for a certain proportion of patients with cervical spondylosis caused by rheumatoid arthritis. Posterior occipito-cervical spinal fusion (OCF) is a common surgical method for the treatment in AAD. But the upper airway obstruction caused by OCF can be life-threatening for patients. We report a patient underwent OCF who developed post-extubation upper airway obstruction in postanesthesia care unit and failed reintubation.

## 2. Case presentation

A 54-year-old female (height,150 cm; body weight, 64 kg) was admitted to the Orthopedics Department because of “repeated multi-joint swelling and pain for 11 + years, which aggravated for 2 weeks.” The admission diagnosis was rheumatoid arthritis and atlanto-occipital deformity. The orthopedic surgeon scheduled a surgery of OCF for her.

**Figure 1.**
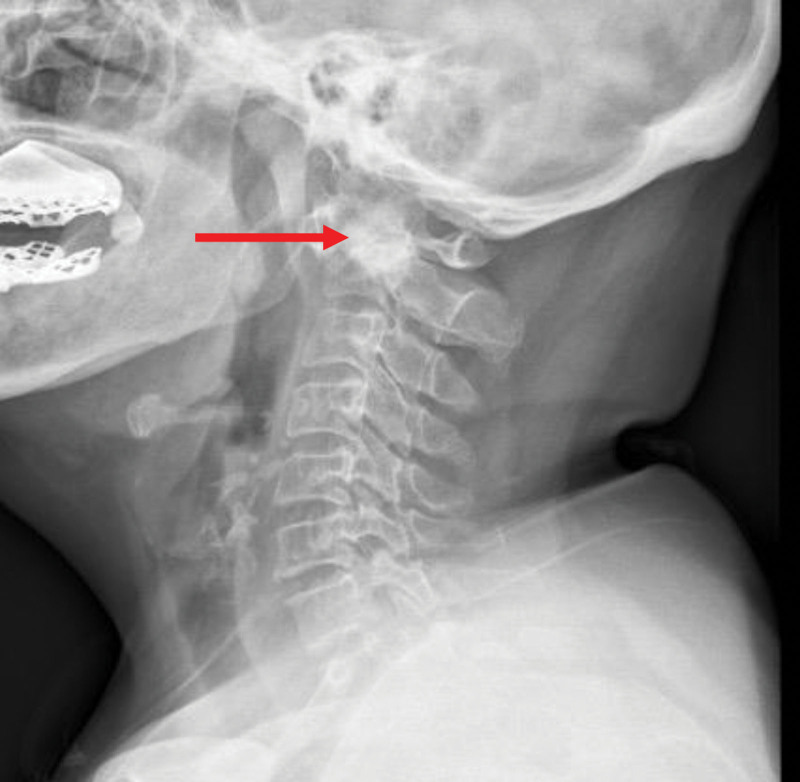
Cervical spine X-ray (lateral view): Atlantoaxial joint space narrowed.

**Figure 2.**
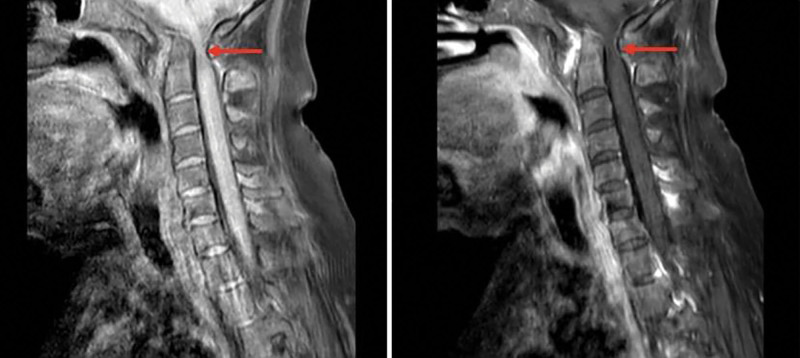
Cervical spine (enhanced) MRI: basilar invagination, plaque-like edema in the marrow; synovium thickening and swelling in the atlantodental space and some intervertebral facet joints, enhanced scan was obviously enhanced, adjacent bone mass erosi. MRI = magnetic resonance.

**Figure 3.**
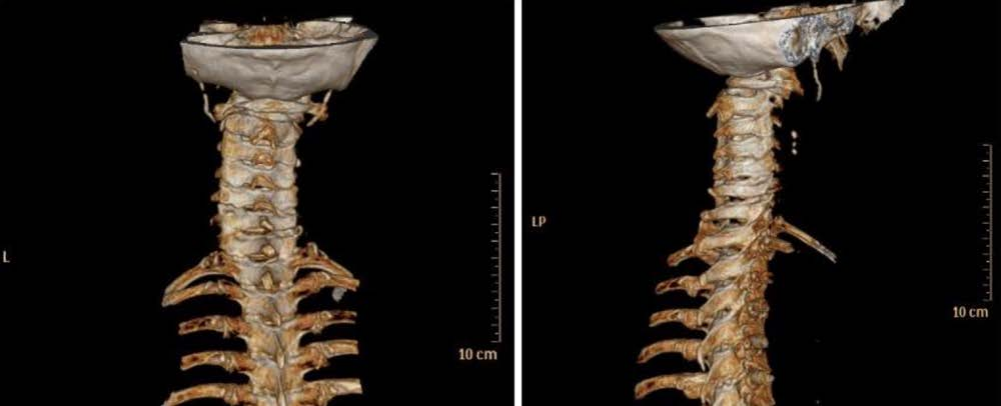
Cervical spine three-dimensional CT: Atlantodontic joint and C1/2 bilateral vertebral surface hyperplasia and sclerosis, local bone resorption and soft tissue shadow under the left vertebral surface of C1/2, corresponding horizontal spinal canal stenosis, atlantoaxial joint subluxation. CT = computed tomography.

A pre-anesthesia visit was completed on the day before the operation: the patient was conscious, bedridden, head and neck movements were limited due to cervical traction, and a mouth opening degree was about 2 cm. Physical examination: She has a chronic illness appearance, there was no positive signs in physical examination of heart, lung and abdomen. She had hypesthesia in the distal extremities of both upper extremities, and her left proximal upper extremity muscle strength was grade 0 to 1 and distal muscle strength was grade 2 to 3. Preoperative examination showed mild anemia (hemoglobin 109 g/L); there was no positive results in blood coagulation test, liver and kidney function, blood sugar and serum electrolyte; erythrocyte sedimentation rate 81 mm/hours; anti-O: 289.0IU/mL, C-reactive protein 35.50 mg/L; anti-cyclic citrullinated peptide antibody > 500.00U/mL. Imaging examination: cervical spine X-ray (lateral view) (as Fig. 1), cervical spine (enhanced) magnetic resonance image (as Fig. 2), cervical spine 3-dimensional computed tomography (as Fig. 3).

Before anesthetics, the standard vital signs were monitored and were as followed: heart rate 91 beats/minutes (bpm), noninvasive blood pressure 155/95 mm Hg, respiratory rate 15 bpm, SpO_2_ 95%. At the same time, a peripheral intravenous channel was established, compound sodium chloride was infused intravenously, and 0.3ug   kg-1   hours-1 of dexmedetomidine was pumped continuously. Under local anesthesia with 1% lidocaine, the left dorsalis pedis artery was punctured and catheterized, then invasive blood pressure monitoring was performed. Intravenous rapid introduction: midazolam 0.05 mg   kg^-1^, sufentanil 0.4ug   kg^-1^, rocuronium bromide 0.6 mg   kg^-1^, propofol 2 mg   kg^-1^. The patient lost consciousness soon, and he was ventilated by mask (tidal volume 400 mL, ventilation frequency 12–16 bpm). Ninety’s later, a 6.5# disposable reinforced endotracheal tube was successfully inserted under the guidance of an oral fiberoptic bronchoscope, the intubation depth was 20 cm. The tracheal catheter was connected to the anesthesia circuit, and the PetCO_2_ waveform was displayed normally. The breath sounds of both lungs were clear and symmetrical during auscultation, and then we fixed the catheter properly. During the surgery, 1% to 2% sevoflurane was inhaled, and 0.1 to 0.2 ug   kg^-1^   minutes^-1^ remifentanil, 0.06 to 0.1 mg   kg^-1^   minutes^-1^ propofol were pumped by intravenous infusion to maintain anesthesia. We adjusted the depth of anesthesia according to her vital signs, and maintained the BIS between 40 to 60 during the operation. The operation lasted 140 minutes. A total of 1500 mL of crystal fluid and 500 mL of colloid fluid were injected into the patient, there was 60 mL of blood loss, and there was 200 mL of urine during the operation. The patient was sent to the postanesthesia care unit with a tracheal tube under spontaneous breathing at the end of operation.

Five minutes later, the patient could shake hands vigorously, and complete the finger-nose test. 1 mg of neostigmine and 0.5 mg of atropine were injection to antagonize non-depolarizing muscle relaxants, and the endotracheal tube was pulled out after sputum suction. Three minutes later, she developed poor ventilation, manifested as upper respiratory obstruction and decreased SpO_2_, and the lowest SpO_2_ was 85%. Immediately we started the difficult airway management process and called the superior physician for help. An oropharyngeal airway was placed in her mouth, and double mask pressure ventilation was given to her. We injected the muscle relaxant antagonist again, maintained the internal environment stable and other symptomatic treatments were taken, but little effect was appeared.

We decided to reintubate for her to save her life. We cleared the sputum and secretions through a fiberoptic bronchoscope, and tried to reintubate. But we failed the reintubation because of the position of the glottis could not be displayed. The cricothyroid membrane was punctured immediately, and high-frequency ventilation was performed to maintain the SpO_2_ at about 80%. Half an hour later, the otolaryngology department successfully performed a tracheotomy, and a ventilation was made for her. After some symptomatic supportive treatment, we sent her to the Intensive Care Unit ward. Postoperative follow-up showed a poor recovery.

## 3. Discussion and conclusion

AAD, which causes a series of dysfunction, is a disease which the atlantoaxial and axial bone articular surfaces lose their normal alignment and stability due to various factors.^[1]^ Among the etiologies, rheumatoid arthritis palys an important role. The most common cervical spine pathologies in rheumatoid arthritis are C1-2 instability (65%), basilar invagination (20%), subaxial subluxation (15%), and combinations of these pathologies.^[2]^ The patient in this case presented as basilar invagination and AAD.

To avoid irreversible neurological damage caused by AAD, early surgery is required. OCF is a classic surgery.^[3]^ A variety of diseases, such as basilar invagination, trauma, tumor, cervical deformity and instability due to rheumatoid arthritis, are indications of OCF.^[4]^ A definitive diagnosis of AAD in our patient, and basilar invagination was showed in magnetic resonance, so OCF was chosen.

However, the incidence of postoperative complications of OCF is relatively high. Zileli M et al^[4]^ followed up 128 patients after OCF from 1994 to 2020, and the average follow-up is 63 months. The results showed that up to 52% of patients experienced postoperative complications. Anesthesia-related important ones of these complications – dysphagia and dyspnea – could have been responsible for the upper airway obstruction and need for reintubation during resuscitation, which was similar to what had happened in this case.

Dysphagia is a common complication after OCF. Hong J et al^[5]^ made a video report on a case of postoperative dysphagia in a patient underwent OCF, whose dysphagia was not resolved until atlantoaxial angle correction surgery was completed. A similar case report was published by Zou Qiang et al,^[6]^ and they analyzed the reason of the postoperative dysphagia was the change in the diameter of the oropharyngeal airway after OCF.

In the study of postoperative airway-related complications, Yoshida M et al^[7]^ reported a case of OCF patients. They found the postoperative occipital-cervical fusion angle (O-C2) changed (from 18° before operation to postoperatively 0°), the posterior pharyngeal wall is thickened, and the middle pharyngeal space is narrowed, resulting in upper airway obstruction in patients. In AADition, Chen Xingjie et al^[8]^ found that patients who underwent posterior atlantoaxial dislocation reduction and internal fixation would experience a reduction in the narrowest distance of the oropharyngeal airway, a reduction in the atlantodental space, and a reduction in the pivotal vertebral angle at the base of the skull, which would cause breathing difficulties and airway obstruction. Sheshadri V et al^[9]^ conducted a retrospective study on 59 patients underwent OCF and found that 16 patients (27%) had airway complications due to postoperative changes in the occipital-cervical angle, of which 4 patients had reintubation. Above all, regardless of postoperative dysphagia or airway complications, the reasons are related to the surgical changes in the occipital-cervical angle.

Combined with the preoperative imaging results of the patient (as Figs. 1–3), and the mouth opening of this patient was limited (2 cm) before operation. The internal fixation was placed during the operation, which severely limited the patient’s head range of head and neck motion. According to the patient’s perioperative fluid input and output (blood loss and urine volume), there was no possibility of throat edema due to excessive fluid load.

To sum up, in AADition to the cervical spondylosis caused by pathological changes, the operation made the airway management more difficult for this patient. Although we completed bronchoscopic-guided orotracheal intubation with adequate preparation before the operation, but we failed reintubation during resuscitation because of the changes in her airway anatomy due to the OCF.

There is some insufficiency in the treatment of this case: We paid insufficient attention for her. When a patient with known difficult airway, it is necessary to prepared for reintubation according to the guidelines^[10]^ during resuscitation and extubation.

The main goal of this case report is to highlight the important of perioperative airway management in these patients who underwent OCF due to cervical spondylosis caused by rheumatoid arthritis. And adequate reintubation preparatory work should be done before extubating at anywhere.

## Author contributions

**Conceptualization:** Yan Weng.

**Data curation:** Yan Wang.

**Funding acquisition:** Qing Zhong.

**Investigation:** Yan Weng.

**Writing – original draft:** Fan Huang.

**Writing – review & editing:** Yan Weng.
